# Changes in metabolic profiles after the Great East Japan Earthquake: a retrospective observational study

**DOI:** 10.1186/1471-2458-13-267

**Published:** 2013-03-23

**Authors:** Masaharu Tsubokura, Morihito Takita, Tomoko Matsumura, Kazuo Hara, Tetsuya Tanimoto, Kazuhiko Kobayashi, Tamae Hamaki, Giichiro Oiso, Masahiro Kami, Tadaichi Okawada, Hidekiyo Tachiya

**Affiliations:** 1 Division of Social Communication System for Advanced Clinical Research, Institute of Medical Science, University of Tokyo, Tokyo, Japan; 2 Baylor Research Institute, Islet Cell Laboratory, Dallas, Texas, USA; 3 Department of Metabolic Disease, Graduate School of Medicine, University of Tokyo, Tokyo, Japan; 4 Department of Hematology and Oncology, JR Tokyo General Hospital, Tokyo, Japan; 5 Department of Hematology and Oncology, Tokyo Metropolitan Bokutoh Hospital, Tokyo, Japan; 6 City Office of Soma, Soma, Fukushima, Japan

**Keywords:** Natural disaster, Health screening, Glycemic control, Lipidemia

## Abstract

**Background:**

A magnitude 9.0 earthquake struck off eastern Japan in March 2011. Many survivors have been living in temporary houses provided by the local government since they lost their houses as a result of the great tsunami (tsunami group) or the expected high-dose radiation resulting from the nuclear accident at the Fukushima Daiichi Nuclear Power Plant (radiation group). The tsunami was more than 9 m high in Soma, Fukushima, which is located 30 km north of the Fukushima Daiichi Nuclear Power Plant and adjacent to the mandatory evacuation area. A health screening program was held for the evacuees in Soma in September 2011. The aim of this study was to compare the metabolic profiles of the evacuees before and after the disaster. We hypothesized that the evacuees would experience deteriorated metabolic status based on previous reports of natural disasters.

**Methods:**

Data on 200 subjects who attended a health screening program in September or October of 2010 (pre-quake) and 2011 (post-quake) were retrospectively reviewed and included in this study. Pre-quake and post-quake results of physical examinations and laboratory tests were compared in the tsunami and radiation groups. A multivariate regression model was used to determine pre-quake predictive factors for elevation of hemoglobin A1c (HbA1c) in the tsunami group.

**Results:**

Significantly higher values of body weight, body mass index, waist circumference, and HbA1c and lower high-density lipoprotein cholesterol levels were found at the post-quake screening when compared with the pre-quake levels (p = 0.004, p = 0.03, p = 0.008, p < 0.001, and p = 0.03, respectively). A significantly higher proportion of subjects in the tsunami group with high HbA1c, defined as ≥5.7%, was observed after the quake (34.3%) than before the quake (14.8%) (p < 0.001). Regional factors, periodic clinic visits, and waist circumference before the quake were identified as predictive factors on multivariate analysis for the deterioration of HbA1c.

**Conclusions:**

Post-quake metabolic variables were impaired compared with pre-quake baseline levels in survivors who were living in temporary houses. A natural disaster could affect metabolic profiles, and careful follow-up for survivors should be planned.

## Background

A historic magnitude 9.0 earthquake devastated the northeast area of Japan on March 11, 2011, resulting in 15,881 deaths and 2,668 missing people according to the Japanese government as of March 2013 [1]. The earthquake was the first world disaster involving a historic tsunami and a nuclear accident, which occurred at the Fukushima Daiichi Nuclear Power Plant and caused low-level radiation exposure to the local residents [2-5]. Most of the evacuees who lost their houses as a result of the tsunami or used to live in the area close to the Fukushima Daiichi Nuclear Power Plant were moved from evacuation shelters to temporary houses provided by the local government by September 2011, 6 months after the earthquake.

Metabolic health problems consequent to natural disasters have been reported. For instance, deteriorated glycemic control was observed after Hurricane Katrina in 2005 [6], and elevated blood pressure was noted after the Hanshin-Awaji Earthquake in 1995 in Japan [7,8]. Thus, the metabolic profiles of the evacuees of the Great East Japan Earthquake in 2011 could be affected by the disaster. To determine their health problems, a health screening program was planned for evacuees who lived in temporary houses in Soma, Fukushima, Japan, in September 2011.

**Figure 1:**
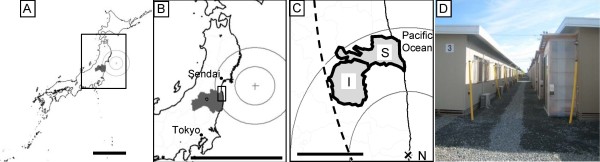
**Geographic details of Soma and Iitate. **Fukushima prefecture (dark area in maps **A** and **B**, where the scale bars indicate 500 km and concentric circles show 100-km intervals) is located southwest of the center of the 2011 Great East Japan Earthquake. Soma City (“S” on the enlarged map **C**, where the scale bar indicates 25 km) is located approximately 170 km from the center of the earthquake (the dotted circle indicates 200 km from the center of the earthquake) and 30 km from the Fukushima Daiichi Nuclear Power Plant (“N” on map **C**, where solid concentric circles indicate 25-km intervals). Soma City experienced an earthquake expressed as a “level 6-low” on the 7-point Japanese seismic intensity scale, indicating that it is difficult for people to remain standing. The tsunami was more than 9.3 m in height in the city. The village of Iitate is on the mountainous side of Soma and in a northwestern direction from the Fukushima Daiichi Nuclear Power Plant (“I” on map **C**). Evacuees are living in temporary prefabricated houses on hills (**D**). The sizes of houses range between approximately 20 m^2 ^for 1 to 3 people and 40 m^2 ^for 4 or 5 people. Evacuees are allowed to live in these houses for up to 3 years after they move in.

Soma City has unique geographic characteristics; it is located on the northeast coast of Japan, close to the center of the earthquake, and approximately 30 km from the Fukushima Daiichi Nuclear Power Plant, near the mandatory evacuation zone where high-level radiation exposure (>20 mSv/y) was expected [9] (Figure 1). The city was engulfed by a tsunami more than 9 m high immediately after the earthquake. According to a report from the local government, there were 475 deaths resulting from the earthquake or tsunami and more than 1,000 buildings were completely damaged in Soma [10]. In addition to the natural disaster of the earthquake and the tsunami, low-level radiation exposure by the Fukushima Daiichi Nuclear Power Plant accident was observed, especially in the mountain area of Soma that is on the leeward side of the plant [2]. Soma experienced a “triple disaster” [4].

The evacuees living in temporary houses in Soma consist of people who moved from the coastal area as a result of the tsunami and those who moved from the mountain area because of high-dose radiation. It has been reported that metabolic disease status could differ based on socioeconomic conditions [11] and that geographic factors are related to diabetes management [12,13]. Hence, the difference in pre-quake residence may have influenced metabolic control during the evacuation because the pre-quake lifestyles of the 2 groups were most likely different; people from the coastal area worked in fishing, sightseeing, or power plants, and those from the mountain area worked in agriculture.

The aim of this study was to compare the metabolic status, before and after the earthquake, of the evacuees who were living in temporary houses in Soma. The results were also compared between evacuees who moved from the coastal area as a result of the tsunami (tsunami group) and those who moved from the mountain area because of the high radiation estimated (radiation group). We hypothesized that metabolic outcomes in the evacuees would be impaired after the quake, as shown in previous reports [6-8].

## Methods

### Study design and participants

**Figure 2:**
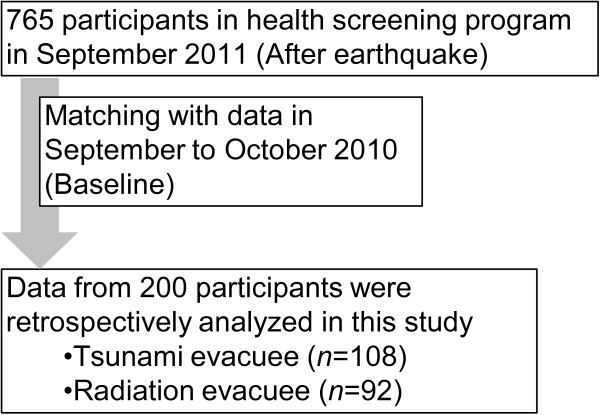
**Flow diagram of subjects. **Data collected at the post-quake health screening were retrospectively analyzed after matching those with pre-quake data.

This retrospective observational study was approved by the ethical review board of the Institute of Medical Science of the University of Tokyo (approval number 23–11). The health screening program for the evacuees living in temporary houses in Soma was conducted from September 19 to 25, 2011. This program was advertised to the residents (n = 2407) by the municipal government of Soma City using bulletin boards in the temporary housing complex approximately 1 month before the screening. Any residents of the temporary housing complex in Soma were able to participate in the health screening program. In total, 765 people aged 1–92 years voluntarily participated in the program. The participants included those who had lived in the districts affected by the tsunami in Soma City (tsunami group) before the earthquake and those who had lived in the village of Iitate, which neighbors Soma City and is located in the mandatory evacuation zone because of radiation exposure (radiation group) (Figure 1) [14]. To compare the health screening data collected before and after the quake, only the participants who were assessed in both September or October 2010 and 2011 (N = 200) were included in this study (Figure 2). There were no interventions in this study.

### Variables in health screening

Height, body weight, waist circumference, blood pressure, and results of blood tests of glucose level, hemoglobin A1c (HbA1c), and lipid panel were examined according to the national guideline that defines comparability and quality of the sample measurement [15]. Body mass index (BMI) was calculated as weight (kg) divided by height squared (m^2^). HbA1c (%) was estimated as the National Glycohemoglobin Standardization Program equivalent value (%) and calculated by the formula HbA1c (%) = 1.02 × HbA1c defined by the Japan Diabetes Society (%) + 0.25%, considering the relational expression of HbA1c measured by the previous Japanese standard substance and measurement methods of the National Glycohemoglobin Standardization Program HbA1c [16].

Reference values were defined based on previous reports or current guidelines for the Japanese population as follows: high BMI, ≥27 kg/m^2^[17]; high waist circumference, ≥85 cm (men) or 90 cm (women) [18]; high systolic/diastolic blood pressure, ≥130/85 mmHg [18]; high HbA1c, ≥5.7% [19]; low high-density lipoprotein (HDL) cholesterol level, ≤1.03 mmol/L (40 mg/dL) [20]; high low-density lipoprotein (LDL) cholesterol level, ≥3.62 mmol/L (140 mg/dL) [20]; and high triglyceride level, ≥1.69 mmol/L (150 mg/dL) [20]. The participants were asked if they visited the clinic for chronic metabolic diseases such as hypertension, cardiovascular disease and diabetes. Medication status was also assessed at the post-quake screening.

### Districts affected by the great tsunami in Soma

Tsunami evacuees were divided into 3 areas according to administrative districts: area 1 was the northeastern area, where the majority of houses or buildings were completely damaged and the residents were mainly employees of the neighboring factories or managed guest houses before the earthquake; area 2 was the eastern area, where houses or buildings were partially damaged and the majority of residents were worked in fishing; and area 3 was the southern area, where the majority of houses were completely destroyed and the livelihood of the residents had been fishing and farming (Additional file 1: Figure S1).

### Statistical analysis

Statistical analysis was performed using IBM SPSS Statistics version 20 (IBM Corporation, Armonk, NY). Data collected in 2010 were used as baseline. Mann–Whitney U or Pearson chi-square tests were performed to compare numerical data or proportions between the tsunami group and the radiation group, respectively. Wilcoxon matched-pair signed-rank test or McNemar’s test was used to compare numerical data or proportions between the 2 years. Subjects were excluded from individual evaluations if they had missing data.

Multivariate logistic regression analysis was used to identify the independent predictive variables at baseline associated with elevated HbA1c between 2010 and 2011, which was defined by a >75% percentile of the distribution. Before multivariate analysis, univariate logistic analysis was performed to select candidates for multivariate analysis. The following variables at baseline were considered potential predictors and examined by univariate analysis: age, gender, residence, regular clinic visits, smoking, body weight, BMI, waist circumference, systemic and diastolic blood pressure, HDL and LDL cholesterol levels, and triglyceride level. The variables with a p value less than 0.10 in univariate models entered multivariate analysis with the backward stepwise technique. Model fit was assessed using the Hosmer-Lemeshow goodness-of-fit test. We considered p values <0.05 as statistically significant. The distribution of values is expressed as median and interquartile interval.

## Results

### Pre-quake characteristics of the participants

The median age of the total cohort (N = 200) was 64 (interquartile interval, 57–71) years in 2010, and there were 81 male (40.5%) and 119 female (59.5%) subjects. The baseline results of health screening are summarized by group in Table 1. The median ages of the subjects in the tsunami and radiation groups were 64 (interquartile interval, 58–69) and 64 (interquartile interval, 55–73), respectively, and there were 70 (64.8%) female subjects in the tsunami group and 49 (53.3%) female subjects in the radiation group. No significant differences in age and the gender ratio were found between the 2 groups. The number of subjects who had missing data is summarized by year and by group in Additional file 2: Table S1.

**Table 1:** Subject characteristics at baseline

**Variables**	**Total cohort (N = 200)**	**Tsunami group (n = 108)**	**Radiation group (n = 92)**	**p value**^**a**^
Age (y)	64 (57–71)	64 (58–69)	64 (55–73)	n.s.
Age >65 y	89 (44.5)	45 (41.7)	44 (47.8)	n.s.
Female gender	119 (59.5)	70 (64.8)	49 (53.3)	n.s.
Physical examination				
Body weight (kg)	58.4 (51.5–64.9)	57.4 (51.8–65.7)	59.3 (51.4–64.4)	n.s.
BMI (kg/m^2^)	24.0 (22.2–25.8)	24.3 (22.1–26.0)	24.3 (22.6–26.0)	n.s.
High BMI	32 (16.2)	17 (15.7)	15 (16.7)	n.s.
Waist circumference (cm)	85.7 (79.8–91.5)	85.8 (80.0–91.6)	85.7 (78.3–90.9)	n.s.
High waist circumference	64 (32.0)	42 (38.9)	22 (23.9)	0.02
Systolic blood pressure (mmHg)	132 (122–144)	132 (126–144)	130 (120–143)	n.s.
High systolic blood pressure	101 (50.5)	57 (52.8)	44 (47.8)	n.s.
Diastolic blood pressure (mmHg)	78 (72–86)	78 (72–86)	79 (70–84)	n.s.
High diastolic blood pressure	51 (25.5)	29 (26.9)	22 (23.9)	n.s.
Laboratory examination				
HbA1c (%)	5.4 (5.1–5.7)	5.2 (5.0–5.4)	5.5 (5.2–5.8)	<0.001
High HbA1c	34 (19.9)	16 (14.8)	18 (28.6)	0.03
HDL cholesterol (mmol/L)	1.50 (1.24–1.81)	1.53 (1.29–1.84)	1.42 (1.20–1.76)	0.04
Low HDL cholesterol	10 (5.0)	3 (2.8)	7 (7.6)	n.s.
LDL cholesterol (mmol/L)	3.08 (2.59–3.67)	3.10 (2.59–3.70)	3.05 (2.61–3.62)	n.s.
High LDL cholesterol	46 (27.1)	31 (28.7)	15 (23.8)	n.s.
Triglyceride (mmol/L)	1.02 (0.69–1.50)	0.94 (0.67–1.33)	1.12 (0.76–1.71)	n.s.
High triglyceride	38 (19.1)	14 (13.0)	24 (26.1)	0.02
Treatment status				
Periodic clinic visits for chronic metabolic diseases	80 (40.0)	44 (40.7)	36 (39.1)	n.s.

Medians and interquartile intervals or number and percentage are shown for numerical or categorical data. High BMI, high waist circumference, high systolic/diastolic blood pressure, high HbA1c, low HDL cholesterol, high LDL cholesterol and high triglyceride were defined by ≥27 kg/m^2^, ≥85 cm (men) or 90 cm (women), ≥130/85 mmHg, ≥5.7%, ≤1.03 mmol/L (40 mg/dL), ≥3.62 mmol/L (140 mg/dL) and ≥1.69 mmol/L (150 mg/dL), respectively, according to the previous reports or current guidelines for the Japanese population.

n.s., not significant.

^a ^Mann–Whitney U or Pearson chi-square tests were performed to compare numerical data or proportions between the tsunami and radiation groups, respectively.

BMI, body mass index; HDL, high-density lipoprotein; LDL, low-density lipoprotein.

A significantly greater population with a high waist circumference was observed in the tsunami group (38.9%) compared with the radiation group (23.9%, p = 0.02); otherwise, no significant differences were found on physical examination. A significantly higher HbA1c, proportion of the population with a high HbA1c, lower HDL cholesterol level, and proportion of the population with a high triglyceride level were observed in the radiation group (p < 0.001, p = 0.03, p = 0.04, and p = 0.02, respectively). Approximately 40% of participants in both groups regularly visited the clinic.

### Post-quake screening results

There was a significant difference in diastolic blood pressure between the tsunami group and the radiation group (p = 0.002, Table 2). In addition, a significantly lower HDL cholesterol level and higher proportion of the population with a low HDL cholesterol level were found in the radiation group compared with the tsunami group. A significantly larger population in the radiation group was treated with medication for chronic metabolic diseases than that in the tsunami group (p = 0.04). There were no significant differences for the other variables between the 2 groups.

**Table 2:** Screening results after the earthquake

**Variables**	**Total cohort**	**Tsunami group**	**Radiation group**	**p value**^**a**^
Physical examination				
Body weight (kg)	58.5 (52.0–66.0)	57.9 (52.6–65.9)	59.5 (51.9–66.3)	n.s.
BMI (kg/m^2^)	24.2 (22.4–26.1)	24.2 (22.1–26.3)	24.3 (22.6–26.0)	n.s.
High BMI	32 (16.2)	17 (15.7)	15 (16.7)	n.s.
Waist circumference (cm)	86.5 (81.9–92.5)	86.0 (81.2–92.5)	87.0 (82.3–92.5)	n.s.
High waist circumference	83 (41.5)	45 (41.7)	38 (41.3)	n.s.
Systolic blood pressure (mmHg)	132 (124–146)	131 (124–144)	134 (126–148)	n.s.
High systolic blood pressure	102 (51.0)	54 (50.0)	48 (52.2)	n.s.
Diastolic blood pressure (mmHg)	80 (70–84)	76 (70–84)	80 (76–86)	0.002
High diastolic blood pressure	48 (24.0)	22 (20.4)	26 (28.3)	n.s.
Laboratory examination				
HbA_1c_ (%)	5.5 (5.4–5.9)	5.5 (5.4–5.9)	5.5 (5.2–5.8)	n.s.
High HbA_1c_	62 (31.0)	37 (34.3)	25 (27.2)	n.s.
HDL cholesterol (mmol/L)	1.47 (1.22–1.76)	1.53 (1.27–1.81)	1.42 (1.14–1.69)	0.02
Low HDL cholesterol	19 (9.5)	6 (5.6)	13 (14.3)	0.04
LDL cholesterol (mmol/L)	3.08 (2.64–3.62)	3.05 (2.59–3.59)	3.13 (2.57–3.66)	n.s.
High LDL cholesterol	48 (24.1)	23 (21.3)	25 (27.5)	n.s.
Triglyceride (mmol/L)	1.00 (0.75–1.57)	1.02 (0.72–1.54)	1.00 (0.78–1.69)	n.s.
High triglyceride	46 (23.1)	23 (21.3)	23 (25.3)	n.s.
Treatment status				
Periodic clinic visits for chronic metabolic diseases	102 (51.0)	50 (46.3)	52 (56.5)	n.s.
Disease for periodic clinic visits				
Hypertension	89 (44.5)	43 (39.8)	46 (50.0)	n.s.
Dyslipidemia	16 (8.0)	7 (6.5)	9 (9.8)	
Diabetes	12 (6.0)	4 (3.7)	8 (8.7)	
Hyperuricemia	3 (1.5)	0 (0.0)	3 (3.3)	
Cardiovascular	11 (5.5)	4 (3.7)	7 (7.6)	
Medication for chronic metabolic diseases	97 (48.5)	45 (41.7)	52 (56.5)	0.04

Medians and interquartile intervals or number and percentage are shown for numerical or categorical data. High BMI, high waist circumference, high systolic/diastolic blood pressure, high HbA1c, low HDL cholesterol, high LDL cholesterol and high triglyceride were defined by ≥27 kg/m^2^, ≥85 cm (men) or 90 cm (women), ≥130/85 mmHg, ≥5.7%, ≤1.03 mmol/L (40 mg/dL), ≥3.62 mmol/L (140 mg/dL) and ≥1.69 mmol/L (150 mg/dL), respectively, according to the previous reports or current guidelines for the Japanese population.

n.s., not significant.

^a ^Mann–Whitney U or Pearson chi-square tests were performed to compare numerical data or proportions between the tsunami and radiation groups, respectively.

BMI, body mass index; HDL, high-density lipoprotein; LDL, low-density lipoprotein.

### Changes in screening data before and after the earthquake

**Figure 3:**
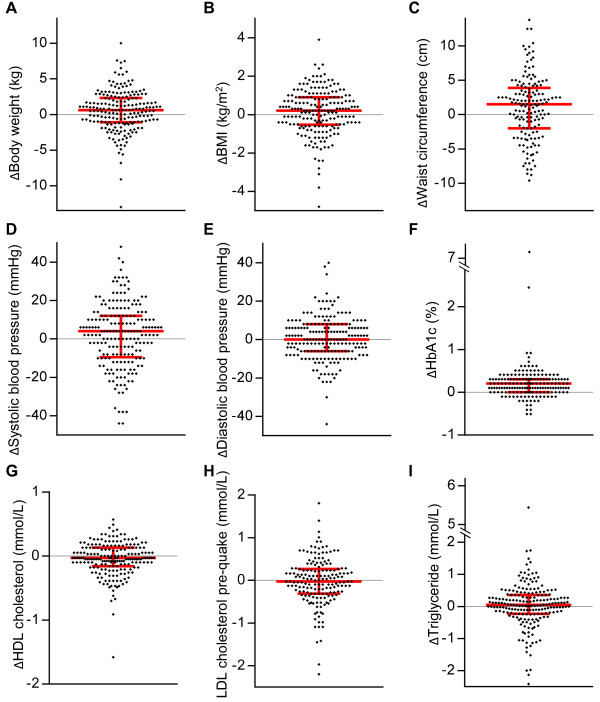
**Scatter plots of differences in the screening variables before and after the earthquake. **Scatter plots of body weight (**A**), BMI (**B**), waist circumference (**C**), systolic blood pressure (**D**), diastolic blood pressure (**E**), HbA1c (**F**), HDL cholesterol (**G**), LDL cholesterol (**H**), and triglyceride (**I**) are shown. Red bars indicate the median and interquartile range.

Changes in health screening variables before and after the earthquake are summarized in Table 3. Significant increases were observed in body weight, BMI, waist circumference, and HbA_1c_ for the total cohort after the earthquake when compared with values before the earthquake (p = 0.004, p = 0.03, p = 0.008, and p < 0.001, respectively), whereas HDL cholesterol levels were significantly decreased (p = 0.03) (Additional file 3: Table S2). For each participant, there were differences in these variables before and after the earthquake (Figure 3). The figure shows that the alteration in each variable was small but was found in the majority of the participants as opposed to significant changes in a small population.

**Table 3:** Changes in health screening variables: post-quake values minus baseline

**Variables**	**Total cohort**	**Tsunami group**	**Radiation group**	**p value**^**a**^
Physical examination				
Body weight (kg)	+0.6 (-1.0, +2.4)^‡^	+0.8 (-1.1, +2.7)^‡^	+0.3 (-1.0, +2.0)	n.s.
BMI (kg/m^2^)	+0.2 (-0.5, +0.9)^†^	+0.2 (-0.6, +0.9)	+0.2 (-0.4, +0.7)	n.s.
Waist circumference (cm)	+1.5 (-2.0, +3.9)^‡^	+0.5 (-3.4, +3.2)	+2.1 (-0.1, +4.5)^‡^	0.03
Systolic blood pressure (mmHg)	+2 (-10, +12)	+2 (-10, +10)	+4 (-4, +13)^‡^	0.03
Diastolic blood pressure (mmHg)	-2 (-7, +6)	-2 (-10, +4)^‡^	+2 (-3, +8)^‡^	<0.001
Laboratory examination				
HbA1c (%)	+0.2 (0.0, +0.3)^‡^	+0.3 (+0.1, +0.4)^‡^	0.0 (-0.1, +0.1)	<0.001
HDL cholesterol (mmol/L)	-0.03 (-0.13, +0.13)^†^	-0.05 (-0.16, +0.10)	0.00 (-0.10, +0.18)	n.s.
LDL cholesterol (mmol/L)	-0.03 (-0.31, +0.26)	-0.04 (-0.31, +0.23)	+0.05 (-0.31, +0.52)	n.s.
Triglyceride (mmol/L)	+0.03 (-0.21, +0.36)	+0.17 (-0.45, 0.92)^†^	-0.01 (-0.30, +0.27)	n.s.
Treatment status				
Periodic clinic visits, new	29 (14.5)	9 (8.3)	20 (21.7)	0.007
Periodic clinic visits, discontinuation	7 (3.5)	3 (2.8)	4 (4.3)	n.s.

Median of change (interquartile range) or number (percentage) are shown.

n.s., not significant.

^a ^Mann–Whitney U or Pearson chi-square tests were performed to compare numerical data or proportions between the tsunami and radiation groups, respectively.

^†^p < 0.05 and ^‡^p < 0.01 for paired comparison between pre-quake and post-quake values using Wilcoxon matched-pair signed-rank test (Additional file 3: Table S2).

BMI, body mass index; HDL, high-density lipoprotein; LDL, low-density lipoprotein.

When comparing these changes between the tsunami and radiation groups, we found significant differences in waist circumference, systolic blood pressure, diastolic blood pressure, and HbA1c (p = 0.03, p = 0.03, p < 0.001, and p < 0.001, respectively; Table 3). Scatter plots of the alterations in the 2 groups are presented in Additional file 4: Figure S2, showing symmetrical distribution. We focused on the change in HbA1c in the tsunami group for multivariate logistic analysis because the lowest p value was observed with this change.

A total of 29 subjects (14.5%) started clinic visits after the earthquake, and significantly more subjects in the radiation group (21.7%) began the visits compared with the tsunami group (p = 0.007). In total, 7 patients discontinued periodic clinic visits.

### Changes in screening data by post-quake treatment status

 The effect of post-quake treatment status of chronic metabolic diseases on the variables measured during screening was determined (Table 4 and Additional file 5: Table S3). The total cohort was divided into 2 groups according to administration of medication for metabolic diseases: medication (N = 97) and no medication (N = 103). A significant change in HDL cholesterol levels was found (p = 0.009), and the other variables did not show significant differences. Twenty-eight of 29 subjects who started periodic clinic visits after the earthquake were treated with medication, and all patients who discontinued clinic visits stopped taking medication (p < 0.001 and p = 0.009, respectively).

**Table 4:** Changes in health screening variables by medication

**Variables**	**Medication group (n = 97)**	**No medication group (n = 103)**	**p value**^**a**^
Physical examination			
Body weight (kg)	+0.3 (-1.2, +1.6)	+0.8 (-0.8, +2.9)^†^	n.s.
BMI (kg/m^2^)	+0.2 (-0.6, +0.7)	+0.3 (-0.4, +0.9)	n.s.
Waist circumference (cm)	+1.5 (-2.0, +4.5)^†^	+1.5 (-2.1, +3.2)	n.s.
Systolic blood pressure (mmHg)	+2 (-12, +14)	+4 (-8, +10)^†^	n.s.
Diastolic blood pressure (mmHg)	-2 (-8, +4)	0 (-7, +6)	n.s.
Laboratory examination			
HbA1c (%)	+0.2 (0.0, +0.4)^‡^	+0.2 (+0.1, +0.3)^‡^	n.s.
HDL cholesterol (mmol/L)	-0.05 (-0.18, +0.10)^‡^	-0.03 (-0.10, +0.13)	0.009
LDL cholesterol (mmol/L)	-0.05 (-0.31, +0.28)	+0.01 (-0.31, +0.24)	n.s.
Triglyceride (mmol/L)	+0.03 (-0.49, +0.65)	+0.03 (-0.46, +0.44)	n.s.
Treatment status			
Periodic clinic visit, new	28 (28.9)	1 (1.0)	<0.001
Periodic clinic visit, discontinuation	0 (0.0)	7 (6.8)	0.009

The alterations in health screening variables were evaluated with post-quake treatment status. Median of change (interquartile range) or number (percentage) is shown.

n.s., not significant.

^a^Mann-Whitney U or Pearson chi-square tests were performed to compare numerical data or ratio data between the tsunami and radiation groups, respectively.

^†^p < 0.05 and ^‡^p < 0.01 for paired comparison between pre-quake and post-quake values using Wilcoxon matched-pair signed-rank test (Additional file 5: Table S3).

BMI, body mass index; HDL, high-density lipoprotein; LDL, low-density lipoprotein.

### Variables predicting change in HbA1c in the tsunami group

We determined predictive variables at baseline for deterioration of HbA1c in the tsunami group. First, the baseline characteristics were tested with the univariate regression model (Table 5). The following 6 factors were identified as candidate factors for multivariate analysis: pre-quake residence in area 2, periodic clinic visits, body weight, BMI, waist circumference, and HDL cholesterol level (p = 0.02, p = 0.008, p = 0.04, p = 0.03, p = 0.07, and p = 0.03, respectively). Interestingly, larger body weight, BMI, and waist circumference were closely linked to deterioration of HbA1c. Multivariate analysis was then used with the 6 candidate factors extracted by the univariate model. Pre-quake residence in area 2, periodic clinic visits, and waist circumference were identified as significant factors in the multivariate model (p = 0.02, p = 0.02, and p = 0.008, respectively) (Table 6).

**Table 5:** Univariate analysis for the prediction of alterations in HbA1c before and after the earthquake

**Variables at baseline**	**Values calculated for OR**	**OR**	**95% CI**	**p value**
Age (year)	1-year increase	1.02	0.97–1.08	0.44
Gender	Men scored as 1	1.69	0.69–4.13	0.25
Residence in area 1	Scored as 1	1.71	0.64–4.61	0.29
Residence in area 2	Scored as 1	0.22	0.06–0.81	0.02
Residence in area 3	Scored as 1	1.69	0.69–4.13	0.25
Periodic clinic visit	Scored as 1	3.40	1.37–8.43	0.008
Smoking	Scored as 1	1.24	0.50–3.09	0.64
Body weight (kg)	1-kg increase	1.05	1.00–1.09	0.04
BMI (kg/m^2^)	1-kg/m^2^ increase	1.17	1.02–1.35	0.03
Waist circumference (cm)	1-cm increase	1.08	1.02–1.14	0.07
Systolic blood pressure (mmHg)	1-mmHg increase	1.02	0.99–1.05	0.323
Diastolic blood pressure (mmHg)	1-mmHg increase	1.03	0.98–1.08	0.26
HDL cholesterol (mg/dL)	1-mg/dL increase	0.96	0.92–0.96	0.03
LDL cholesterol (mg/dL)	1-mg/dL increase	1.00	0.99–1.02	0.86
Triglyceride (mg/dL)	1-mg/dL increase	1.00	0.996–1.01	0.39

Odds ratios (OR), 95% confidential intervals (CI), and p values for baseline variables in univariate logistic regression models to predict changes in HbA_1c_ before and after the earthquake are shown. The variables with p values < 0.1 were entered in a multivariate model. Gender; regional factors of area 1, 2, and 3; periodic clinic visits; and smoking were treated as categorical values (score of 0 or 1) and the other variables as continuous values. BMI, body mass index; HDL, high-density lipoprotein; LDL, low-density lipoprotein.

**Table 6:** Predictors of the change in HbA1c in tsunami evacuees before and after the earthquake

**Baseline variables**	**OR**	**95% CI**	**p value**
Residence in area 2	0.17	0.04–0.71	0.02
Periodic clinic visits	3.36	1.25–9.06	0.02
Waist circumference (cm)	1.08	1.02–1.15	0.008

Odds ratios (OR), 95% confidential intervals (CI), and p values for baseline variables in multivariate logistic regression models to predict changes in HbA_1c_ before and after the earthquake are shown. The subjects were scored as 1 if they lived in area 2 or periodically visited clinics before the quake. The OR for waist circumference was calculated corresponding to the 1-cm increase. The Hosmer-Lemeshow goodness-of-fit test was not significant (p = 0.991), indicating good fit.

## Discussion

The results of this study showed that the metabolic variables of body weight, BMI, waist circumference, HbA1c, and HDL cholesterol level deteriorated in evacuees living in temporary houses in Soma, Fukushima, based on comparison of pre-quake and post-quake records. This finding suggests that chronic metabolic health problems such as diabetes and hypercholesterolemia should be carefully monitored and treated after a natural disaster, although acute diseases of infection and psychological issues are also major issues in the health and wellness of survivors in the early phase of disaster relief [3,21].

Impaired HbA1c, HDL cholesterol levels, and triglyceride levels were observed in the radiation group at baseline when compared with the tsunami group, although post-quake screening showed that HDL cholesterol was the only significantly different laboratory test result between the 2 groups. A significant increase in HbA1c and triglyceride levels was found in the tsunami group. Thus, the historic disaster was more likely to have a negative effect on metabolic laboratory test results in the tsunami group than in the radiation group. This difference might be related to changes in exercise and meals in the subjects’ daily lives before and after the quake [22,23]; however, direct evidence could not be shown in this report because data on exercise and meals were rarely collected before the earthquake.

Findings on physical examination such as body weight, BMI, waist circumference, and blood pressure did not largely change after the earthquake, although statistically significant changes were found in body weight, BMI, and waist circumference in the total cohort. The differences are seen in the majority of the cohort on the scatter plots. A previous report showed that blood pressure could be elevated for 1 or 2 weeks after a major earthquake but return to pre-quake levels 1 month later [24], which is consistent with our findings in the total cohort because the post-quake health screening was conducted 6 months after the earthquake. However, in the radiation group, a significant elevation of systolic and diastolic blood pressure was observed at post-quake screening. Mental stress triggered by the nuclear accident could be a cause of these changes in the radiation group because psychological stress could affect blood pressure [25]. Meanwhile, glycemic control was worsened in the total cohort and the tsunami group after the earthquake. Inui *et al.* demonstrated that HbA1c was elevated and peaked 3 to 4 months after the Hanshin-Awaji earthquake in January 1995 and that changes in psychological status and lifestyle possibly influenced the metabolic disease [26].

The proportion of patients who visited the clinic periodically for chronic metabolic diseases was not significantly different between the tsunami and radiation groups before the earthquake; however, after the earthquake, a significantly higher proportion of participants in the radiation group were seen in the clinic. A total of 21.7% of the participants in the radiation group started clinic visits after the earthquake compared with 8.3% in the tsunami group. Eighty-five percent of the participants in the radiation group who were newly followed up by physicians after the earthquake had hypertension, and all were treated with medication (data not shown). These findings suggest that hypertension commonly develops after a natural disaster and that alterations in lifestyle might affect the onset [24,25].

We also determined the effect of medication on screening outcomes. Similar changes were seen regardless of whether the participants were treated with medication or not, although there was a significant difference in HDL cholesterol levels; significant impairment was found in the medication group. We did not collect detailed information on medications, such as drug name and dose, so we may have missed the influence of a particular drug in the screening results. This is a limitation of this study, and further investigation is required to address the effect of medication on the evacuees.

Baseline variables of residence, periodic clinic visits, and waist circumference were identified as independent predictors for the change in HbA1c in the tsunami group, which had the lowest p value on statistical evaluation. Regular clinic visits and waist circumference should be considered potential risk factors for diabetes; however, interestingly, regional information was included in the prediction model. The reason of regional differences might be related to psychosocial changes as a result of the property damage, which can be related to glycemic control [26] and employment status because employment could affect the development of diabetes [27]. Local company employees might have resumed their jobs if their companies could recover their workplace, but fishing and farming families have not been able to restart their jobs because of radiation contamination. Further investigations including social background are required to explore the causes of the changes in health screening variables.

Currently, local physicians have initiated home visiting medical care services, suggesting a considerable solution for health problems in temporary housing [28]. The number of heath care providers dramatically decreased after the earthquake, resulting in the closure of hospitals. Medical resources in Fukushima prefecture are limited, with 192.5 physicians per 100,000 compared with the national average of 223.9 before the earthquake [29]. Increasing the number of health care professionals is an urgent issue in the disaster area.

This study has several limitations; a small cohort with a limited age range, use of retrospective analysis, short follow-up period and lack of information on social background that may cause restrained statistical power or unknown bias. The participants in this study might have high concern for health wellness, which suggests that the risk of developing metabolic diseases shown in this study might be lower than that in the whole population living in the temporary housing complex. Only 32% of residents of the temporary housing complex in Soma (765 of 2407 residents) participated in the health screening in 2011, although no exclusion criteria were defined. In addition, caution is needed when the results from this study are extended to other disasters because only a portion of the evacuees was examined in this study. Further and long-term investigation with a larger cohort should be planned in the future.

## Conclusions

 In summary, post-quake variables of BMI, waist circumference, HbA1c, and HDL cholesterol level were impaired compared with pre-quake levels in survivors who lived in temporary houses in Soma, Fukushima. The natural disaster of a big earthquake could result in deteriorated metabolic profiles, and long-term medical follow-up should be carefully planned for evacuees.

## Competing interests

The authors declare that they have no competing interests.

## Authors’ contributions

MTS, MT, TM, and MK designed the study, performed data collection, and contributed to writing the manuscript. TT, KK, TH, GO, TO, and HT contributed to planning the health screening program and data collection. MTS and MT performed statistical analysis and wrote the draft. KH contributed to scientific review. All authors read and approved the final manuscript.

## Pre-publication history

The pre-publication history for this paper can be accessed here:

http://www.biomedcentral.com/1471-2458/13/267/prepub

## Supplementary Material

Additional file 1: Figure S1 Districts suffered from the great tsunami in Soma. The districts in Soma that mentioned in this article are presented with aerial photographs before and after the great earth quake. Click here for file

Additional file 2: Table S1 Number of missing data in each group. The numbers of subjects who have missing data are shown. Click here for file

Additional file 3: Table S2 Paired comparison of metabolic data before and after the earthquake. p values for the comparison of screening variables before and after the earthquake are shown when the subjects are divided by the tsunami and radiation groups. Click here for file

Additional file 4: Figure S2 Scatter plots of the screening variables by groups. The scatter plots are presented to show the alternations in screening variables before and after the quake by the tsunami and radiation groups. Click here for file

Additional file 5: Table S3 Paired comparison of metabolic data before and after the earthquake by post-quake treatment status. p values for the comparison of screening variables before and after the earthquake are shown when the subjects are grouped by post-quake treatment status. Click here for file
