# Supplementary material for: Changes in metabolic profiles after the Great East Japan Earthquake: a retrospective observational study
Source: BMC Public Health. 2013 Mar 23;13:267. doi: 10.1186/1471-2458-13-267 (PMC3614525; doi:10.1186/1471-2458-13-267)
Supplement: Additional file 1: Figure S1 — Districts suffered from the great tsunami in Soma. The districts in Soma that mentioned in this article are presented with aerial photographs before and after the great earth quake. [file 1471-2458-13-267-S1.pdf]

Supplemental Figure 1. Districts suffered from the great tsunami in Soma.

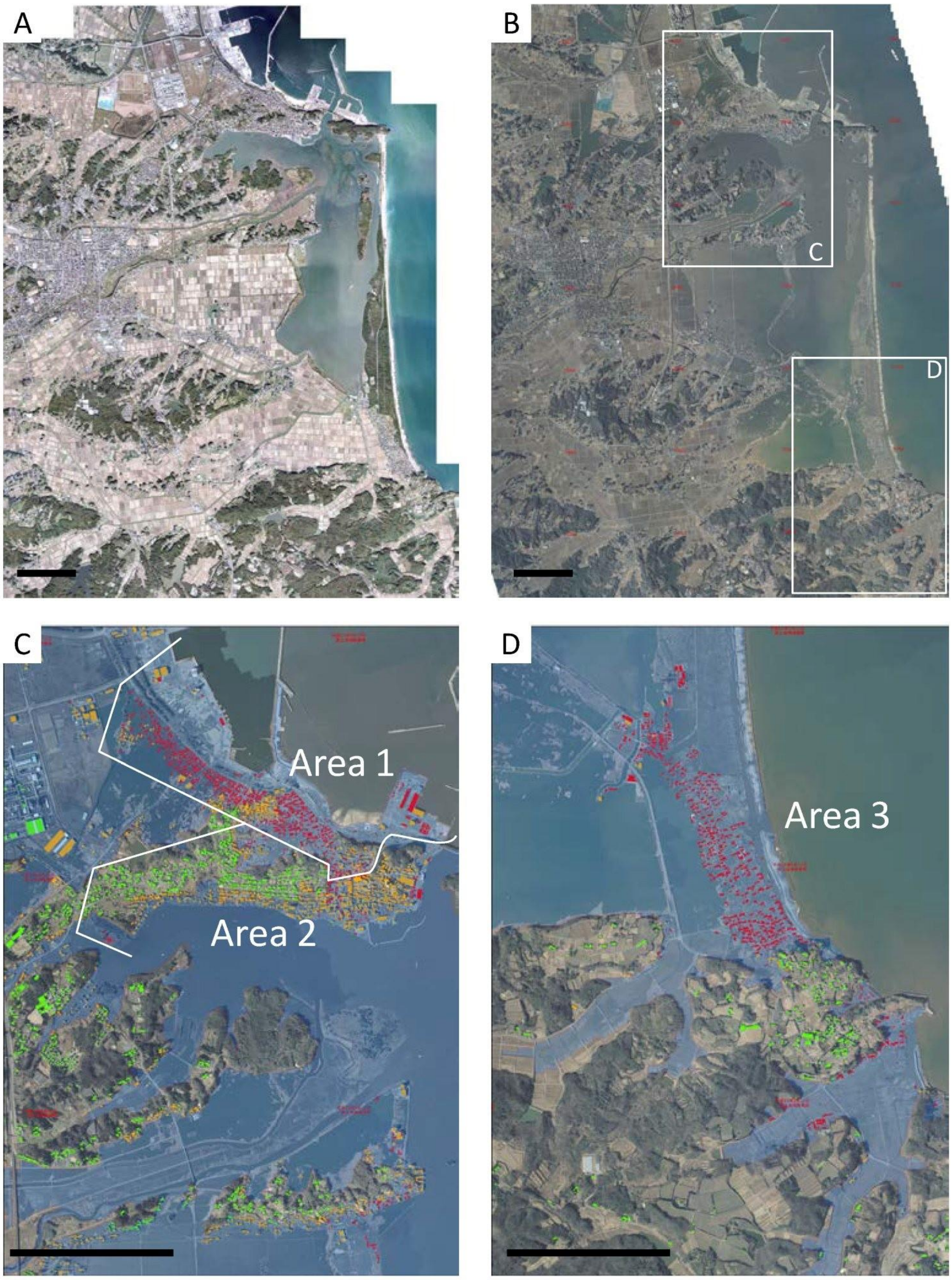

Aerial photographs before (in 2010, A) and after (on March 14th 2011, B) the great earthquake are shown. The degree of property damage is shown as complete (red), half (yellow) and partial (green) damage (Figure C and D). Areas 1 to 3 were defined based on the administrative districts in Soma city. Area 1: northeastern area where majority of houses or buildings were completely damaged and the residents were mainly

company employees of the neighbor power plant or managed guest houses before the earthquake, Area 2: eastern area where houses or buildings were partially damaged and the majority of residents were fishing families, and Area 3: southern area where the majority of houses were completely destroyed and the residents had lived on fishing and farming. Scale bars in all figures indicate 1 km.
