# Supplementary material for: Changes in metabolic profiles after the Great East Japan Earthquake: a retrospective observational study
Source: BMC Public Health. 2013 Mar 23;13:267. doi: 10.1186/1471-2458-13-267 (PMC3614525; doi:10.1186/1471-2458-13-267)
Supplement: Additional file 2: Table S1 — Number of missing data in each group. The numbers of subjects who have missing data are shown. [file 1471-2458-13-267-S2.doc]

Supplemental Table 1. Number of missing data in each group.

| Variables | Total cohort | | Tsunami group | | Radiation group | |
| --- | --- | --- | --- | --- | --- | --- |
| Age | 0 | | 0 | | 0 | |
| Sex | 0 | | 0 | | 0 | |
|  |  | |  | |  | |
| Physical examination | 2010 | 2011 | 2010 | 2011 | 2010 | 2011 |
| Body weight | 0 | 0 | 0 | 0 | 0 | 0 |
| BMI | 0 | 0 | 0 | 0 | 2 | 0 |
| Waist circumstance | 47 | 2 | 4 | 0 | 43 | 2 |
| Systolic blood pressure | 0 | 0 | 0 | 0 | 0 | 0 |
| Diastolic blood pressure | 0 | 0 | 0 | 0 | 0 | 0 |
| Laboratory examination |  |  |  |  |  |  |
| HbA1c | 29 | 0 | 0 | 0 | 29 | 0 |
| HDL cholesterol | 0 | 1 | 0 | 0 | 0 | 1 |
| LDL cholesterol | 29 | 1 | 0 | 0 | 29 | 1 |
| Triglyceride | 0 | 1 | 0 | 0 | 0 | 1 |

The numbers of subjects who have missing data are shown.
