# Supplementary material for: Changes in metabolic profiles after the Great East Japan Earthquake: a retrospective observational study
Source: BMC Public Health. 2013 Mar 23;13:267. doi: 10.1186/1471-2458-13-267 (PMC3614525; doi:10.1186/1471-2458-13-267)
Supplement: Additional file 3: Table S2 — Paired comparison of metabolic data before and after the earthquake. p values for the comparison of screening variables before and after the earthquake are shown when the subjects are divided by the tsunami and radiation groups. [file 1471-2458-13-267-S3.doc]

Supplemental Table 2. Paired comparison of metabolic data before and after the earthquake.

| Variables | Total cohort | Tsunami group | Radiation group |
| --- | --- | --- | --- |
| Physical examination |  |  |  |
| Body weight | 0.004 | 0.006 | *n.s.* |
| BMI | 0.03 | *n.s.* | *n.s.* |
| High BMI—proportion | *n.s.* | *n.s.* | *n.s.* |
| Waist circumference | 0.008 | *n.s.* | 0.001 |
| High waist circumference —proportion | 0.004 | *n.s.* | 0.002 |
| Systolic blood pressure | *n.s.* | *n.s.* | 0.005 |
| High systolic blood pressure—proportion | *n.s.* | *n.s.* | *n.s.* |
| Diastolic blood pressure | *n.s.* | 0.009 | 0.001 |
| High diastolic blood pressure—proportion | *n.s.* | *n.s.* | *n.s.* |
| Laboratory examination |  |  |  |
| HbA1c | < 0.001 | <0.001 | *n.s.* |
| High HbA1c—proportion | <0.001 | <0.001 | *n.s.* |
| HDL cholesterol | 0.03 | *n.s.* | *n.s.* |
| Low HDL cholesterol—proportion | 0.02 | *n.s.* | *n.s.* |
| LDL cholesterol | *n.s.* | *n.s.* | *n.s.* |
| High LDL cholesterol—proportion | *n.s.* | *n.s.* | *n.s.* |
| Triglyceride | *n.s.* | 0.04 | *n.s.* |
| High Triglyceride—proportion | *n.s.* | *n.s.* | *n.s.* |

**p* values are shown when Wilcoxon matched-pair signed rank or McNemar test were performed to compare paired numerical data or ratio between pre and post-quake.
