# Supplementary figures and images for: Changes in metabolic profiles after the Great East Japan Earthquake: a retrospective observational study
Source: BMC Public Health. 2013 Mar 23;13:267. doi: 10.1186/1471-2458-13-267 (PMC3614525; doi:10.1186/1471-2458-13-267)

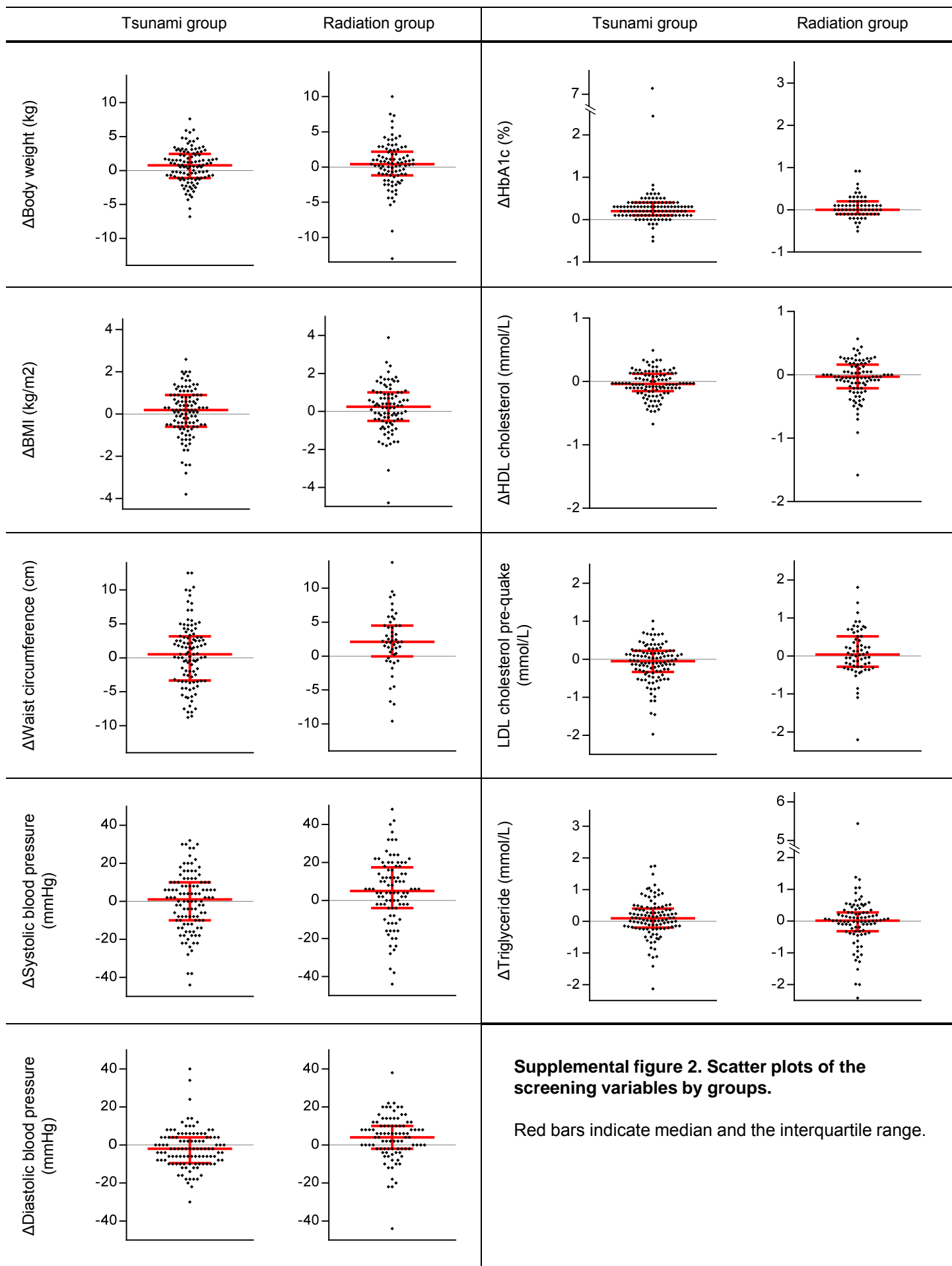

Supplement: Additional file 4: Figure S2 — Scatter plots of the screening variables by groups. The scatter plots are presented to show the alternations in screening variables before and after the quake by the tsunami and radiation groups. [file 1471-2458-13-267-S4.pdf]
