# Supplementary material for: Changes in metabolic profiles after the Great East Japan Earthquake: a retrospective observational study
Source: BMC Public Health. 2013 Mar 23;13:267. doi: 10.1186/1471-2458-13-267 (PMC3614525; doi:10.1186/1471-2458-13-267)
Supplement: Additional file 5: Table S3 — Paired comparison of metabolic data before and after the earthquake by post-quake treatment status. p values for the comparison of screening variables before and after the earthquake are shown when the subjects are grouped by post-quake treatment status. [file 1471-2458-13-267-S5.doc]

Supplemental Table 3. Paired comparison of metabolic data before and after the earthquake by post-quake treatment status.

| Variables | Medication group | No medication group |
| --- | --- | --- |
| Physical examination |  |  |
| Body weight | *n.s.* | 0.02 |
| BMI | *n.s.* | *n.s.* |
| Waist circumference | 0.02 | *n.s.* |
| Systolic blood pressure | *n.s.* | 0.02 |
| Diastolic blood pressure | *n.s.* | *n.s.* |
| Laboratory examination |  |  |
| HbA1c | <0.001 | <0.001 |
| HDL cholesterol | 0.002 | *n.s.* |
| LDL cholesterol | *n.s.* | *n.s.* |
| Triglyceride | *n.s.* | *n.s.* |

**p* values are shown when Wilcoxon matched-pair signed rank or McNemar test were performed to compare paired numerical data or ratio between pre and post-quake. Abbreviation; *n.s.*: not significant
